# Bottom‐up rather than top‐down mechanisms determine mesocarnivore interactions in Norway

**DOI:** 10.1002/ece3.11064

**Published:** 2024-03-07

**Authors:** Rocío Cano‐Martínez, Neri Horntvedt Thorsen, Tim R. Hofmeester, John Odden, John Linnell, Olivier Devineau, Siow Yan Jennifer Angoh, Morten Odden

**Affiliations:** ^1^ Department of Forestry and Wildlife Management Inland Norway University of Applied Sciences Koppang Norway; ^2^ Norwegian Institute for Nature Research Oslo Norway; ^3^ Department of Wildlife, Fish and Environmental Studies Swedish University of Agricultural Sciences Umeå Sweden; ^4^ Norwegian Institute for Nature Research Lillehammer Norway

**Keywords:** camera trap, carnivore community, intraguild interactions, Norway, structural equation modeling

## Abstract

Interactions among coexisting mesocarnivores can be influenced by different factors such as the presence of large carnivores, land‐use, environmental productivity, or human disturbance. Disentangling the relative importance of bottom‐up and top‐down processes can be challenging, but it is important for biodiversity conservation and wildlife management. The aim of this study was to assess how the interactions among mesocarnivores (red fox *Vulpes vulpes*, badger *Meles meles*, and pine marten *Martes martes*) were affected by large carnivores (Eurasian lynx *Lynx lynx* and wolf *Canis lupus*), land cover variables (proportion of agricultural land and primary productivity), and human disturbance, as well as how these top‐down and bottom‐up mechanisms were influenced by season. We analyzed 3 years (2018–2020) of camera trapping observations from Norway and used structural equation models to assess hypothesized networks of causal relationships. Our results showed that land cover variables were more strongly associated with mesocarnivore detection rates than large carnivores in Norway. This might be caused by a combination of low density of large carnivores in an unproductive ecosystem with strong seasonality. Additionally, detection rates of all mesocarnivores showed positive associations among each other, which were stronger in winter. The prevalence of positive interactions among predators might indicate a tendency to use the same areas and resources combined with weak interference competition. Alternatively, it might indicate some kind of facilitative relationship among species. Human disturbance had contrasting effects for different species, benefiting the larger mesocarnivores (red fox and badger) probably through food subsidization, but negatively affecting apex predators (wolf and lynx) and smaller mesocarnivores (pine marten). In a human‐dominated world, this highlights the importance of including anthropogenic influences in the study of species interactions.

## Graphical abstract

We used 3 years of camera trap data from Norway to assess how the interactions among mesocarnivores (red fox, badger, and pine marten) were affected by large carnivores (Eurasian lynx and wolf), land cover variables, and human disturbance, as well as how these top‐down and bottom‐up mechanisms were influenced by season. Our results showed that land cover variables were stronger predictors of mesocarnivore activity than large carnivores in Norway. Human disturbance had contrasting effects for large carnivores and mesocarnivores. Humans can influence species through different top‐down and bottom‐up processes, which highlights the complexity of anthropogenic effects on species interactions.

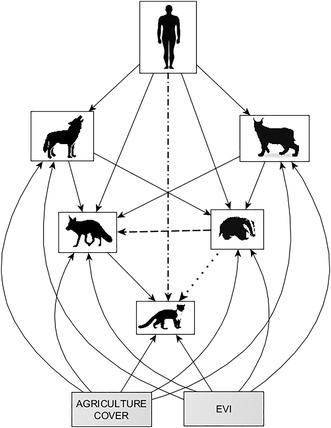

## 1 INTRODUCTION

The study of interspecific interactions among mammalian carnivores is fundamental to conservation biology (Linnell & Strand, 2000), but the factors influencing these interactions can be complex and difficult to disentangle. The potential top‐down effect of large carnivores and their importance in maintaining ecosystem functioning have been widely recognized (Estes et al., 2011; Ripple et al., 2014; Ritchie & Johnson, 2009). This top‐down regulation has been lost in many regions due to the decline of large carnivores caused by human persecution and habitat loss, which has led to changes in species interactions and food webs (e.g., “mesopredator release”, Crooks & Soulé, 1999), highlighting their ecological role in ecosystems worldwide (Ripple et al., 2014). Even though negative interspecific interactions among carnivores seem to be widespread, there is also a growing recognition of the importance of positive interactions in structuring predator communities (Prugh & Sivy, 2020; Sirén et al., 2021). Large carnivores can facilitate mesocarnivores by providing resource subsidies in the form of carrion (Pereira et al., 2014; Prugh & Sivy, 2020), which can be important food sources in areas where several opportunistic mesocarnivores coexist (Sivy et al., 2018), or when other food sources are scarce (Jedrzejewski & Jedrzejewska, 1992; Killengreen et al., 2011). Interactions between large carnivores and mesocarnivores can therefore range from facilitation to suppression, and both may even occur simultaneously. For instance, carcasses may act as an extra food source for mesocarnivores, but they can also increase aggressive encounters between the two groups (see “fatal attraction hypothesis”; Prugh & Sivy, 2020; Sivy et al., 2017). In addition, the direction and strength of these interactions may be scale and context dependent (Sivy et al., 2017; Wikenros et al., 2017).

Interactions among predators in human‐dominated landscapes can be different from those occurring in undisturbed habitats. Furthermore, ecological phenomena such as mesopredator release can be difficult to separate from land‐use changes (Prugh et al., 2009). Anthropogenic influence on food webs may operate through diverse processes and influence multiple trophic levels simultaneously. For example, humans can decrease predator density directly through hunting, but they can also trigger behavioral responses at both spatial and temporal scales (e.g., by changing predators' habitat use and activity patterns) (Milner et al., 2007; Ordiz et al., 2012, 2021). These behavioral responses, in turn, might cause top‐down cascades that may affect species at lower trophic levels. On the other hand, humans may also influence predators through bottom‐up processes via food subsidization (Gompper & Vanak, 2008; Newsome et al., 2014, 2015), or by enhancing forage availability for herbivores, thus increasing prey density (Muhly et al., 2013). The effect of such food subsidies may be particularly relevant for systems with low productivity (Melis et al., 2009).

Ecosystem productivity and seasonal change in resource availability can determine the relative strength and direction of trophic interactions (Elmhagen & Rushton, 2007; Ritchie & Johnson, 2009; Stoessel et al., 2018). Previous studies have found that, in ecosystems with low productivity and harsh winter conditions, bottom‐up factors exert a stronger control on species interactions than top‐down factors (Elmhagen & Rushton, 2007; Sirén et al., 2021; Stoessel et al., 2018). These studies highlight the importance of considering both top‐down and bottom‐up processes when studying mesopredator interspecific interactions in ecosystems with strong seasonality. Unfortunately, large‐scale experimental approaches that might help disentangle the relative importance of top‐down versus bottom‐up effects are rarely logistically possible (Nilsen et al., 2020). Modeling approaches based on large‐scale observational data can be a useful approximation (Dorresteijn et al., 2015; Elmhagen & Rushton, 2007). The relevance of such insights is of special importance in the face of climate change, which will alter seasonal conditions and subsequently species interactions (Montoya & Raffaelli, 2010).

Several large carnivore species are now recovering across large parts of Europe (Chapron et al., 2014), with potential cascading effects through the entire carnivore community. In Southern Spain, for example, the recovery of the Iberian lynx (*Lynx pardinus*) exerted a strong suppression control of two sympatric mesocarnivores (Burgos et al., 2023). However, the strength of this top‐down effect differed for different mesocarnivore species, and it was modulated by prey availability. In Scandinavia, the recovery of Eurasian lynx (*Lynx lynx*) and wolves (*Canis lupus*) in human‐modified ecosystems is raising a key question regarding the ecological role that large carnivores play in these anthropogenic landscapes and their impacts on mesocarnivores relative to the effects of humans (Dorresteijn et al., 2015; Kuijper et al., 2016).

The aim of this study was to assess how small‐scale spatial interactions among mesocarnivores were affected by large carnivores, land cover variables (proportion of agricultural land and primary productivity), and human disturbance. The study also aimed to investigate how the relative strength of these top‐down and bottom‐up mechanisms was influenced by season (summer vs. winter) over a large spatial scale. To achieve this, we used 3 years of data from a national‐level camera trapping study in Norway to assess species interactions by calculating their detection rates. We used a carnivore guild that included Eurasian lynx and wolves as apex predators, red foxes (*Vulpes vulpes*) and badgers (*Meles meles*) as dominant mesocarnivores, and pine martens (*Martes martes*) as a subordinate mesocarnivore.

The recovery of large carnivore populations in Scandinavia, combined with climate warming, might strengthen top‐down factors (Sirén et al., 2021; Stoessel et al., 2018). There is some evidence that the recovering lynx populations in Scandinavia significantly limit mesocarnivore populations, such as red fox, in some areas (Elmhagen et al., 2010; Fedriani et al., 1999; Helldin et al., 2006; Pasanen‐Mortensen et al., 2013). Under this scenario, we predicted that large carnivores (lynx and wolf) would have a negative effect on the larger mesocarnivores (red fox and badger) through interference competition, resulting in an indirect net benefit to the small predator (pine marten). However, as previously found in ecosystems with low productivity, bottom‐up factors might have a stronger influence on the carnivore community structure than top‐down factors, especially in winter due to resource constraints (Elmhagen & Rushton, 2007; Stoessel et al., 2018). Under this other scenario, we expected that the proportion of agricultural land and primary productivity would have a stronger effect on mesocarnivore detections rates than large predators. Regarding the influence of humans, mesocarnivores are strongly influenced by anthropogenic food supplies, and red fox and badger densities have been found to be higher, and their home ranges smaller, in urban and suburban areas compared to semi‐natural habitats (Šálek et al., 2015). Thus, we predicted a positive effect of humans on red fox and badger detection rates through food subsidization, resulting in an indirect negative effect on pine marten through intraguild predation (Lindström et al., 1995).

## 2 MATERIALS AND METHODS

### 2.1 Study area

**FIGURE 1:**
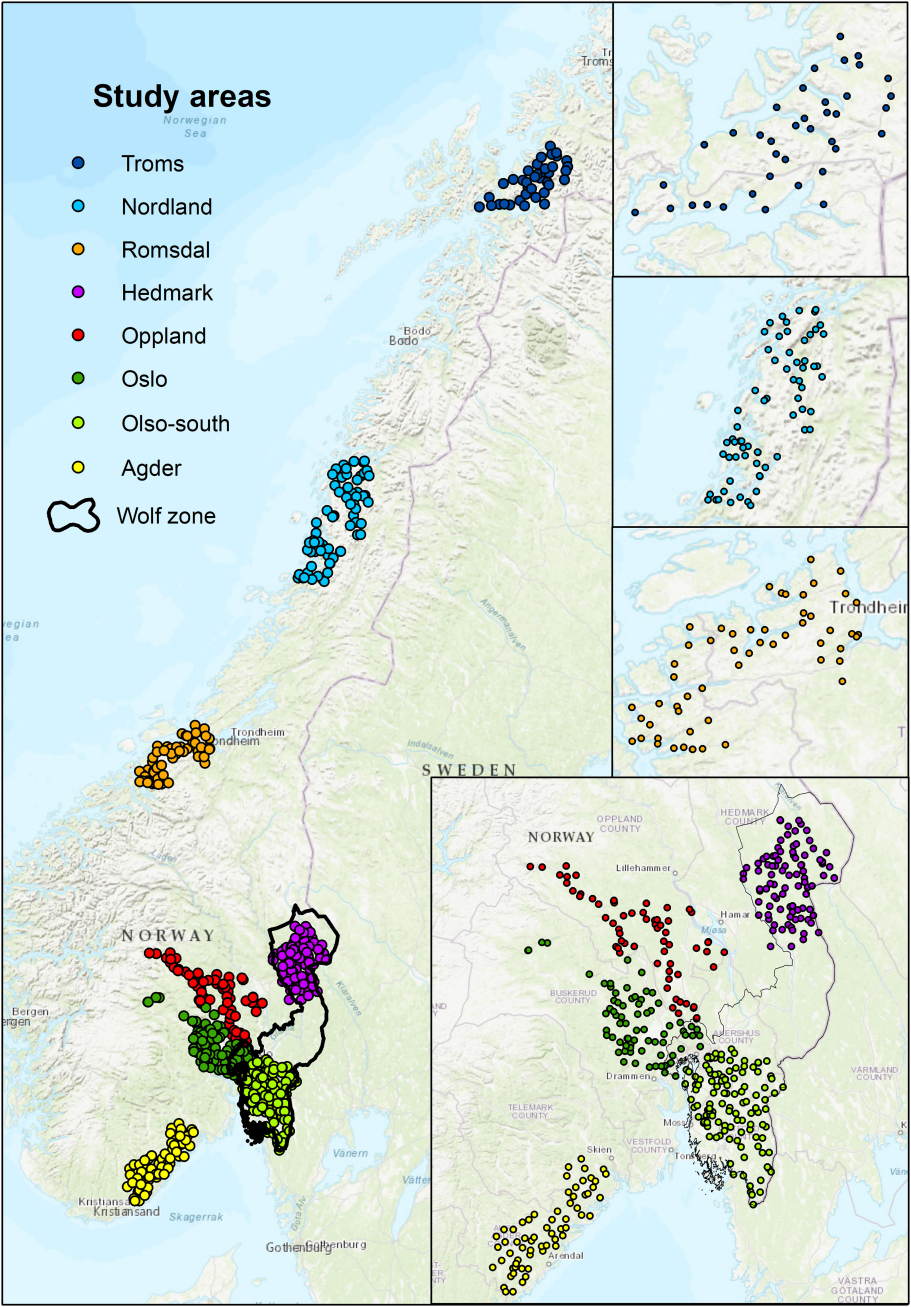
Location of the camera traps in Norway, study areas based on geographic clusters of camera traps, and the wolf zone.

We conducted this study within nine different counties in Norway, representing a gradient of landscape productivity and human influence from Troms & Finnmark County in the north (68° N), to Agder County in the south (58° N) (Figure 1). The southern areas are in general characterized by more fragmented habitats, with forested areas intermixed with agricultural fields and scattered human settlements, and high productivity, whereas the northern areas are characterized by continuous habitats, with boreal forests and alpine tundra, and are less productive. Forests are dominated by conifers, mainly Scots pine (*Pinus sylvestris*) and Norway spruce (*Picea abies*), intermixed with deciduous species such as birch (*Betula pubescens* and *B. pendula*), rowan (*Sorbus aucuparia*), aspen (*Populus tremula*), gray alder (*Alnus incana*), and willow (*Salix caprea*), which are more abundant in the south. Mean annual temperatures decrease with latitude, being milder in the south (annual mean temperature 7.8°C in Kristiansand) than in the north (annual mean temperature – 0.2°C in Tromsø) (no.climate‐data.org), and winter severity (i.e. snow depth and low temperatures) increases with latitude and altitude. Human population densities range from 1642.7 inhabitants per km^2^ in Oslo County to 3.4 inhabitants per km^2^ in Troms & Finnmark (www.ssb.no). Lynx can potentially be found across the entire study area but exhibit higher densities in the southernmost areas. Wolves are restricted to the wolf management zone in southeastern Norway (Figure 1), where wolf density was 0.004 wolves/km^2^ in the winter of 2022/2023 (Svensson et al., 2023). Wolf density outside the wolf management zone is practically zero, as wolves outside the zone are culled as part of management plans.

### 2.2 Camera trapping

We used 3 years of data (2018–2020) from 618 camera trap locations as part of an ongoing long‐term camera trapping study (SCANDCAM project, viltkamera.nina.no). The SCANDCAM project has volunteer‐run camera traps (HC500, HC600, PC800, PC850, PC900, and HP2X, Reconyx, Holmen, Wisconsin, USA) to monitor lynx family groups. One camera trap was typically placed within 50 km^2^ grid cells (although in a few cases two or more camera traps were placed in a single grid cell), covering 30,950 km^2^ in Norway (Figure 1). The minimum distance among camera traps was 3983 m (25% quantile: 1934 m; 75% quantile: 5796 m). To maximize the probability of detecting lynx and other predators, local volunteers in cooperation with trained technicians placed the cameras preferably on forest roads, trails, or natural movement routes for wildlife. Each camera trap was placed 60–120 cm above the ground and aimed at the landscape feature of interest. All camera traps were programmed with the most sensitive sensor setting to take one photo when triggered by an animal passing, as well as a daily time‐lapse image at 8 a.m., in order to check if the unit functioned correctly and if the field of view was clear. Memory cards and batteries were switched at least four times a year. A deep convolutional neural network trained with previous images from the SCANDCAM project was used to classify all images using TensorFlow (Abadi et al., 2016). All species identifications were in addition manually verified by trained staff and students. All images of humans and vehicles were automatically removed to conform to Norwegian privacy regulations, but we retained information of their passing. A detailed explanation of the pre‐processing and classification workflow can be found in Hofmeester et al. (2021).

We calculated species detection rate as the number of days in which an animal (lynx, wolf, red fox, badger or pine marten) was detected by a camera trap per year and season, corrected for camera effort (i.e., number of days during which the camera trap was active). This detection rate results from a combination of both local density and activity of predators (Carbone et al., 2001). This is useful for our study because it not only reflects the relative abundance of species present but also the intensity of use of a specific area.

### 2.3 Covariates

All covariates were extracted in ArcGIS (ESRI, 2014). We used a Norwegian and a Swedish vegetation map merged together to account for the cameras along the border (Northern Research Institute's vegetation map, Norway, 30 × 30 m resolution merged with Swedish Corine land cover map Lantmäteriet, Sweden, 25 × 25 m resolution into a 25 × 25‐m resolution raster, Mattisson et al., 2013; Ordiz et al., 2015), from which we extracted the proportion of agricultural land (i.e. arable land and pastures) and the proportion of forest. We assessed local human disturbance at camera sites by calculating the number of days in which humans were detected per year and season, corrected for camera effort. As an additional measure of human disturbance, we obtained human density (inhabitants/km^2^) from Statistics Norway as a 250 m resolution raster (www.ssb.no). However, since most of the cameras were placed outside urban areas, with no humans at the grid cell, we used the human density raster to calculate a proxy for the direct influence of human disturbance at the camera sites. Specifically, we created a point layer from the human density raster and predicted a planar kernel density map set with bandwidth = 2000 and cell size = 200 m. We then extracted proportion of agricultural land, proportion of forest, and human disturbance to a 1‐km radius circular buffer around each camera trap. We selected this scale in order to capture enough variation of the landscape around each camera trap than would be possible with a smaller radius, while still maintaining a value that allowed the assessment of small‐scale species interactions rather than species distribution.

In order to assess primary productivity, we downloaded the 1‐km resolution monthly enhanced vegetation index (EVI) from the MOD13A3 V061 product (Didan, 2021) using the AppEEARS application (AppEEARS‐Team, 2022). We downloaded monthly data for years 2016–2020 and deleted 11 raster cells because of low quality and missing data. We then merged the monthly EVI map layers into one final raster containing an average value of overlapping cells. We masked out water to include only terrestrial cells and calculated the mean‐EVI for a 10‐km radius circular buffer around each camera trap. We used a 10‐km buffer for primary productivity in order to focus on the wider region rather than on the direct surroundings of the camera site. Using an environmental productivity variable as a covariate such as EVI can help to correct for biases when considering multiple sites (Hofmeester et al., 2019).

The number of parameters used in a SEM is limited by the sample size (Grace, 2006). To avoid overfitting the model, we decided to use two seasons: from October to March (winter; roughly the months with snow cover) and from April to September (summer; snow free months in most of the study area). These seasons (winter vs. summer) allowed us to reflect the broadest and most dramatic seasonal shifts in this environment. We also considered variation among geographic clusters of camera traps, which we included as an 8‐level covariate called “study area” (Figure 1).

### 2.4 Data analysis

All statistical analyses were carried out in R version 3.6.1 (R‐Core‐Team, 2019). We standardized (scaled) all continuous covariates by subtracting the mean and dividing by one standard deviation. We used Pearson's correlation tests to check for collinearity among continuous variables, with a limit of r ≤ 0.6 (Zuur et al., 2010). Preliminary analysis indicated that the covariates proportion of forest and proportion of agricultural land were highly correlated and behaved similarly. Therefore, we decided to include the proportion of agricultural land, which better reflects human impacts. We also evaluated which “human” covariate (i.e., local records from camera traps, or human disturbance) provided a better model fit according to k‐fold cross‐validation values, and we included human disturbance in the final model.

We used structural equation modeling (SEM) to test how interactions among mesocarnivores (red fox, badger and pine marten) were affected by large carnivores (wolf and lynx), land cover variables (proportion of agricultural land and EVI), and human disturbance, as well as how these interactions were affected by season (summer vs. winter). SEM provides a multivariate framework to develop and evaluate hypothesized networks of causal relationships, estimating the relative strength of direct and indirect paths within the system (Grace, 2006; Grace et al., 2012). SEMs are built by single equations that represent hypothetical relationships (i.e., pathways) between variables, which are identified a priori based on documented knowledge of the study system. In these equations, the response variables in one equation may form predictors in others, thereby forming sequences of casual relationships that are structured in a single piecewise model (Grace, 2006).

#### 2.4.1 Model description

**FIGURE 2:**
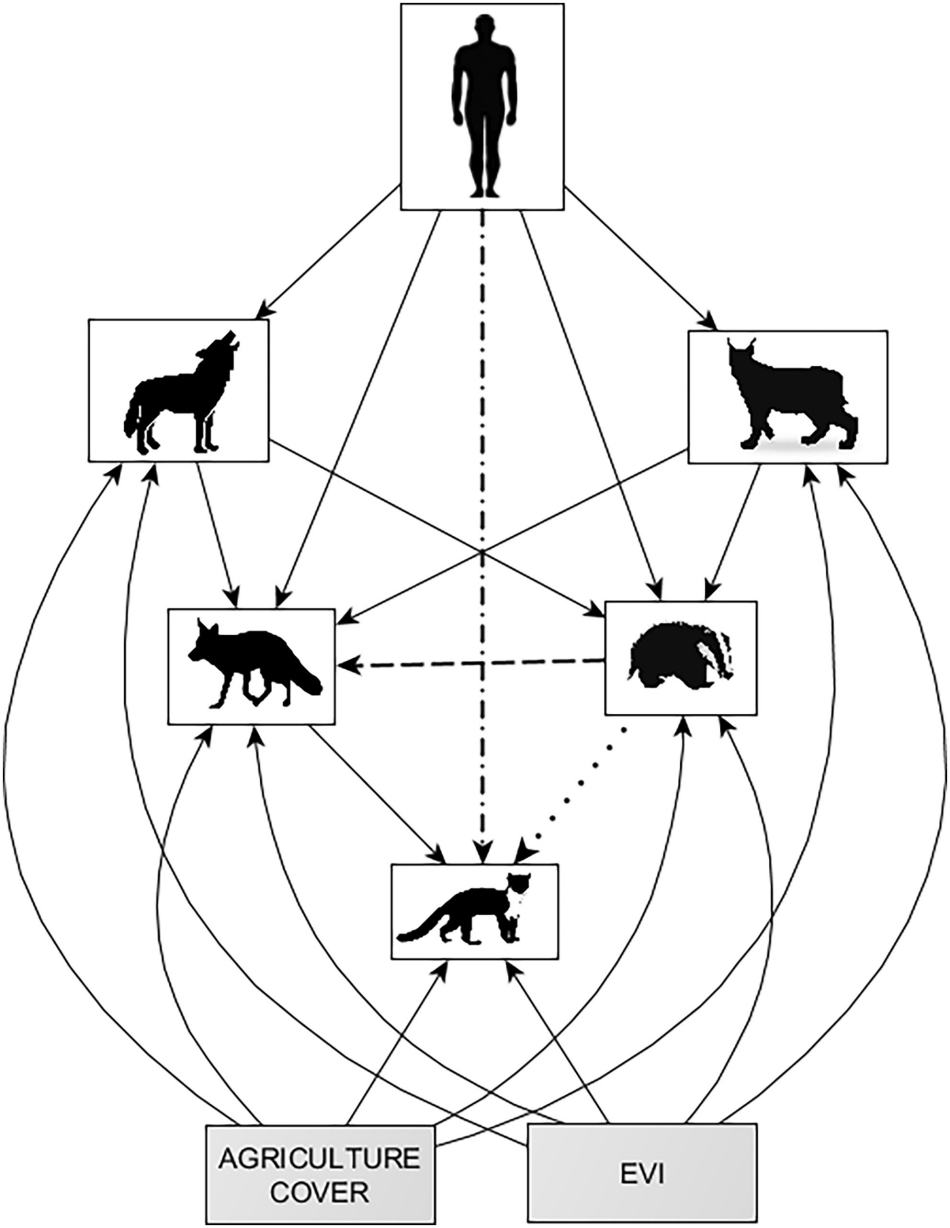
A priori structural equation models describing all hypothesized top‐down and bottom‐up interactions evaluated. Model 1 (baseline model) is represented by solid lines between variables. Models 2, 3, and 4 are as Model 1, but with added paths from badger to red fox (Model 2, dashed line), from badger to pine marten (Model 3, dotted line), and from humans to pine marten (Model 4, dashed‐dotted line).

We considered top‐down and bottom‐up pathways based on our predictions and on documented predator interactions in boreal ecosystems (solid lines in Figure 2, Model 1). Large carnivores (lynx and wolf) were expected to limit the dominant mesocarnivores (red fox and badger) through interference competition (Elmhagen & Rushton, 2007). Dominant mesocarnivores, red foxes, were expected to limit pine martens through the same process (Zalewska et al., 2021). We expected that bottom‐up factors (proportion of agricultural land and EVI) would have a positive effect on all species. However, we expected a seasonal difference, with a stronger effect of bottom‐up factors on species interactions in winter than in summer relative to the effect of top‐down factors (Elmhagen & Rushton, 2007; Stoessel et al., 2018). Previous studies have found low interference competition between wolf and Eurasian lynx (Schmidt et al., 2009; Wikenros et al., 2012). Therefore, we anticipated no interference between these two large carnivores in our study area. Humans were expected to limit large carnivores through disturbance and culling (Dorresteijn et al., 2015), and to have a positive effect on dominant mesocarnivores due to food subsidization (Gompper & Vanak, 2008).

Additionally, we proposed three other alternative models to test against Model 1. This is because there is a large potential for intraguild competition between badger and the other two mesocarnivores (red fox and pine marten). The three of them can occupy similar ecological niches, share the same den sites (Macdonald et al., 2004; Mori et al., 2015) and, being opportunistic generalists, may share foods such as earthworms, smaller vertebrates, eggs, and fruits (Kauhala et al., 1998; Macdonald, 1980; Prigioni et al., 2008; Torretta et al., 2016). However, differential use of time and space may enable coexistence (Zalewska et al., 2021). Therefore, we added an additional pathway from badger to red fox (Model 2 in Figure 2) and then a second pathway from badger to pine marten (Model 3 in Figure 2). Moreover, pine martens are considered to avoid urban areas (Fusillo et al., 2009; Goszczyński et al., 2007), although recent research suggests that they can adapt to live in areas with anthropogenic disturbances (Weber et al., 2018). Therefore, we added a third pathway from humans to pine marten (Model 4 in Figure 2).

#### 2.4.2 SEM analysis

To construct the SEM, we used generalized linear mixed models with a negative binomial distribution to model overdispersion, using the Bayesian *brms* package version 2.19.0 (Bürkner, 2017). We used the number of species detections (number of days in which an animal was detected by a camera per year and season) as the response variable, with the log‐transformed number of active camera trap days as an offset. Depending on the species, explanatory standardized variables included: (i) large carnivores (wolf and lynx) detections and human disturbance, and (ii) land cover variables (proportion of agricultural land and EVI). We allowed the model to estimate different values for each standardized pathway for winter and summer by adding the interaction of season with all pathways. We also included the study area and camera location as random intercepts.

We included the default flat priors of brms and fitted the models using 3000 iterations on 3 chains. We used leave‐one‐out cross‐validation (LOO) from the loo package to estimate the expected log‐posterior density (ELPD), a (relative) measure of predictive error, which can be used to compare models similar to the AIC criterion (Vehtari et al., 2017). We checked convergence by looking at the trace plots of the MCMC chains and with the Gelman and Rubin convergence diagnostic *Ȓ* (Gelman & Rubin, 1992). We also calculated a Bayesian *R* ^2^ (Gelman et al., 2018) for the best fitting model using the bayes_R2 function in the *brms* package (Bürkner, 2017) to assess the variance explained by the main factors and present standardized path coefficients in order to compare across all pathways. Below, we present the posterior median and associated 90% credible interval (CRI) for all parameters, which we discuss in terms of non‐overlapping CRI for convenience. We also present the Probability of Direction (*pd*) index of effect existence (Makowski et al., 2019), defined as the proportion of the posterior distribution of the same sign as the median, that is, the (un)certainty that an effect is either positive or negative. We also report the Region Of Practical Equivalence (ROPE; e.g., Kruschke, 2014), which assesses the magnitude and importance of an effect (e.g., if % in ROPE <2.5%, the null hypothesis that the slope = 0 is rejected; if % in ROPE >97.5%, the coefficient is “equivalent” to zero and the null hypothesis cannot be rejected) (Makowski et al., 2019).

## 3 RESULTS

In 3 years of camera trapping (1095 days with observations across 618 trap locations), 99.5% of days we obtained pictures of red foxes (50.4% in summer and 49.5% in winter), 97.7% of humans (51.1% in summer and 48.8% in winter), 84.6% of badgers (59.3% in summer and 40.7% in winter), 60.8% of pine martens (65.2% in summer and 34.8% in winter), 56.3% of lynx (45.2% in summer and 54.8% in winter), and 35.4% of wolves (57.7% in summer and 42.3% in winter).

Of the 618 camera trap locations, 81.4% detected red fox in summer and 73.6% in winter; 60.5% detected badger in summer and 43.0% in winter; 35.1% detected pine marten in summer and 25.1% in winter; 23.6% detected lynx in summer and 23.7% in winter; and 11.5% detected wolf in summer and 11.2% in winter.

We tested the baseline model (Model 1, solid lines in Figure 2) with three other models by adding additional pathways. We selected Model 4, which included all the tested pathways, as the best fitting model (Table 1). Bayesian *R* ^2^ values for Model 4 were 0.53 for red fox, 0.64 for badger, 0.55 for pine marten, 0.45 for lynx, and 0.55 for wolf.

**TABLE 1:** Comparison of the four tested models, ranked by relative predictive accuracy (Expected Log‐Posterior Density, elpd_diff), and associated uncertainty (se_diff). Model 4 is the best, as per Vehtari et al. (2017) and (Sivula et al., 2020). Model 1 corresponds to the baseline model, to which an extra pathway was added for each of models 2–4.

	elpd_diff	se_diff	Added pathways
Model 4	0.00	0.00	Humans → Pine marten Badger → Pine marten Badger → Red fox
Model 3	−2.23	4.03	Badger → Pine marten Badger → Red fox
Model 2	−23.06	6.75	Badger → Red fox
Model 1	−77.99	10.38	Baseline

Below, we discuss coefficients whose 90% CRI did not overlap zero, but we also present the *pd* and ROPE for some of the main pathways to ease interpretation. All estimates of posterior distributions, *pd* and ROPE can be found in Appendices 1, 2, 3.

### 3.1 Top‐down versus bottom‐up effects

**FIGURE 3:**
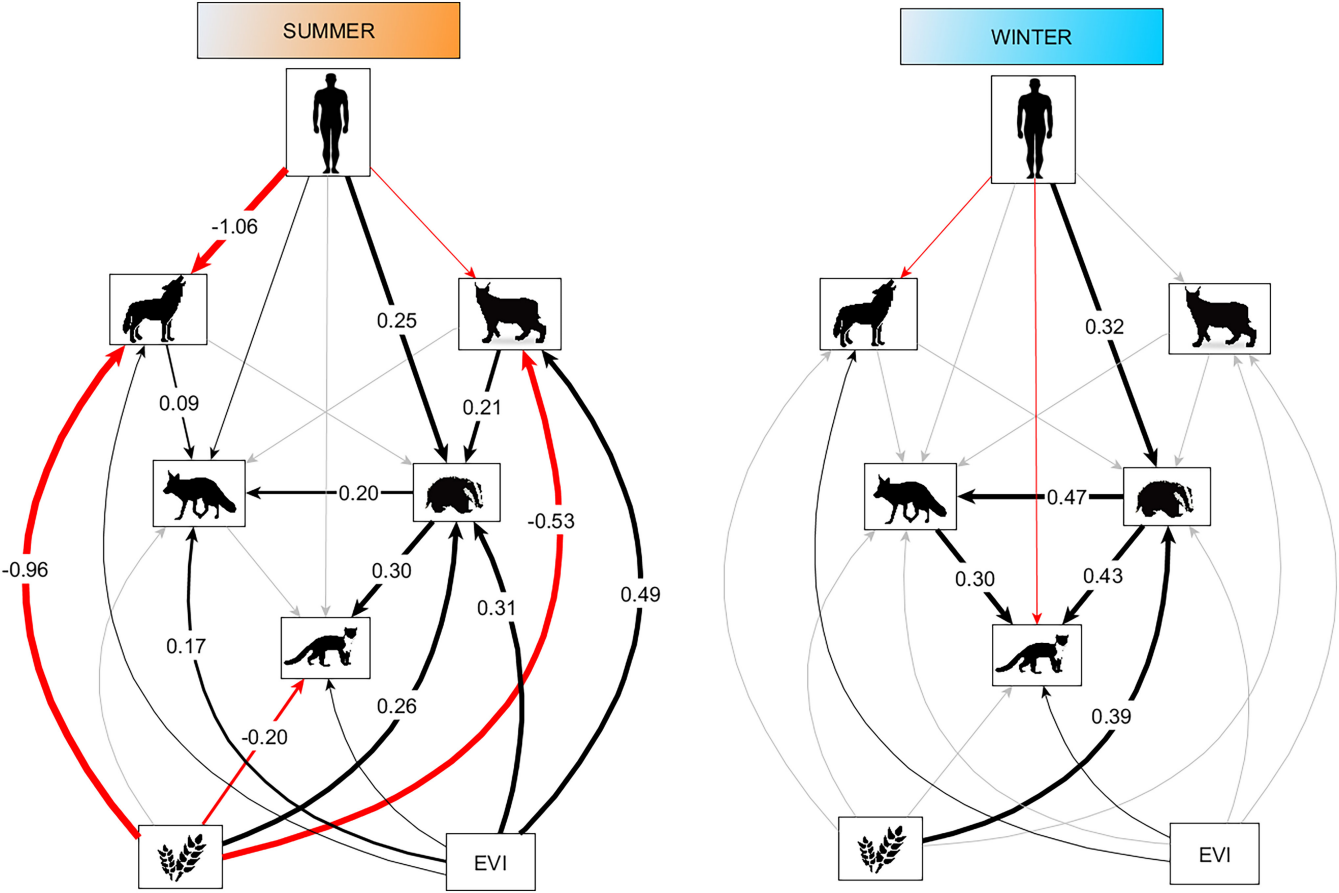
Structural equation model (SEM) with the best fit showing top‐down and bottom‐up pathways for large carnivores and mesocarnivores. We allowed the model to estimate different values for each pathway for each season. Therefore, we show one model for summer (left figure) and another model for winter (right figure). Values along arrows represent the relative magnitudes of positive (black) and negative (red) standardized path coefficients whose 90% credible interval (CRI) did not overlap zero. Arrows with no coefficients represent those pathways included in the model whose 90% CRI overlap 0. However, we still indicate positive (black) and negative (red) for those pathways that are discussed in the text given their significant Probability of Direction (*pd*) and Region of Practical Equivalence (ROPE) values. Gray arrows represent pathways included in the model whose 90% CRI overlap 0 but with very weak evidence according to their *pd* and ROPE.

There was a positive relationship between lynx and badger in summer, although the proportion of agricultural land and EVI were stronger predictors of badger detection rate (Figure 3). There was also a positive association between wolf and red fox detection rates in summer (Figure 3), but this relationship was quite weak (58% of the posterior distribution was in the ROPE; Appendix 3), and red fox detection rate was more associated with badger presence and EVI (Figure 3). Both red fox and badger detection rates were stronger predictors of pine marten detection rates than land cover variables in winter (Figure 3). Indeed, all mesocarnivores (red fox, badger and pine marten) showed positive interactions among each other, which were stronger in winter (Figure 3).

Land cover variables had contrasting effects for different species and seasons. The proportion of agricultural land was a positive predictor for badger detection rates in both seasons, and a negative predictor for pine martens, wolves, and lynx in summer (Figure 3). Additionally, EVI was a positive predictor of lynx, red fox, and badger detection rates, but only in summer (Figure 3). There was also some evidence for a positive association between EVI and wolf detection rates, even though the CRI for the median overlapped 0, only 13% and 5% of the posterior distribution was in the ROPE in summer and winter, respectively; and *pd* = 0.87 in summer and pd = 0.97 in winter indicates a 87% and a 97% chance that the effect of EVI on wolf detection rates was positive in summer and winter, respectively (Appendix 3). Similarly, we observed some evidence of a positive association between EVI and pine marten detection rates, although weak; even though the CRI for the median overlapped 0, 39% and 49% of the posterior distribution was in the ROPE in summer and winter, respectively; and *pd* = 0.71 in summer and *pd* = 0.70 in winter (Appendix 3).

### 3.2 The effect of human disturbance

Human disturbance had a positive association with badger detection rates, which was slightly stronger in winter (Figure 3). There was also some evidence for a positive association between humans and red fox detection rates in summer, although weak; even though the CRI for the median overlapped 0, 49% of the posterior distribution was in the ROPE, and *pd* = 0.94 (Appendix 1). On the other hand, there was some evidence for a negative association between human disturbance and pine marten detection rates, which was more certain in winter (*pd* = 0.75 in summer and *pd* = 0.99 in winter); although the CRI for the median overlapped 0, only 6% of the posterior distribution was in the ROPE in winter (Appendices 1 and 3). Human disturbance had a strong negative association with wolf detection rates, which was more certain in summer (*pd* = 0.96 in summer and *pd* = 0.76 in winter) (Figure 3, Appendix 3). There was also a high probability that the association between humans and lynx would be negative in summer; even though the CRI for the median overlapped 0, only 18% of the posterior distribution was in the ROPE, and *pd* = 0.91 (Appendix 3).

## 4 DISCUSSION

Disentangling the relative importance of bottom‐up and top‐down processes is important for biodiversity conservation and wildlife management (Elmhagen & Rushton, 2007). Our results suggest that bottom‐up processes (land cover variables) were stronger predictors of mesocarnivore activity than top‐down processes (large carnivores) in Norway. We also observed a predominance of positive associations between species, which were stronger in winter among the mesocarnivores and probably linked to the use of similar areas and resources. Human disturbance, on the other hand, presented contrasting effects for different species at different trophic levels, showing a positive association with red fox and badger, and a negative association with wolf, lynx and pine marten.

### 4.1 Predominance of bottom‐up over top‐down mechanisms

Mesocarnivores in our study area seemed to be more influenced by bottom‐up rather than by top‐down mechanisms, as the effect of large carnivores was weak compared to the effect of land cover variables. Both agricultural land and EVI were important predictors of species detection rates, although we found no association between the proportion of agricultural land and red fox detection rates, probably because of their ability to adapt to a wide variety of habitat types. This predominance of bottom‐up over top‐down factors has been previously observed in similar ecosystems with low productivity and strong seasonality, where environmental constraints seem to have a stronger influence on the community structure than the presence of competitors (Elmhagen & Rushton, 2007; Stoessel et al., 2018; Wikenros et al., 2017). Large carnivores in Norway may also occur at too low densities to have a significant effect on the detection rates of mesocarnivores at a large scale. In Norway, lynx and wolf populations are actively managed via culling to limit their density and to constrict their distribution (Linnell et al., 2010). This is reflected in the negative association observed between humans and large carnivores in our study. A reduced predator population exposed to hunting may not be able to play its full ecological role, limiting both direct and indirect effects on other predators and prey (Ordiz et al., 2013). In such a system, it is predicted that bottom‐up influence will be stronger than top‐down control of mesocarnivores (Pasanen‐Mortensen & Elmhagen, 2015). This implies that the abundance of smaller carnivores would be dependent on prey availability, which ultimately is related to bioclimatic factors such as environmental productivity and land use (Pasanen‐Mortensen & Elmhagen, 2015). However, climate change might cause a switch from bottom‐up to top‐down regulation of ecosystems through enhanced primary productivity (Legagneux et al., 2014). This can have a significant impact in the regulation of ecosystems in northern regions.

### 4.2 Predominance of positive associations among species

There was a predominance of positive associations among species. Large carnivores had a positive effect on mesocarnivore detection rates, with a positive association between lynx and badger and between wolf and red fox in summer (although weaker for the last pair). A previous study by Sivy et al. (2017) reported local‐scale positive associations between wolves and red foxes (among other non‐apex predators), in Canada, which they attributed to carrion facilitation. Carcasses left by apex predators present a risky yet a predictable food source that could benefit smaller predators (Prugh & Sivy, 2020; Selva et al., 2005; Wikenros et al., 2014). The slight positive effect of wolf on red fox detection rates in summer (which included spring months in our analysis) could be related to food provisioning during reproduction, when species have a higher energy demand, as suggested by previous studies in Sweden (Wikenros et al., 2013, 2014). On the other hand, the positive association between lynx and badgers could be due to the bigger size and higher aggressiveness of badgers with respect to other mesocarnivores. This may cause lynx to avoid badgers, resulting in less aggressive interactions between these two carnivore species. In fact, there are no reports of aggressive interactions between these two carnivores (Neal & Chesseman, 1996, as cited in Fedriani et al., 1999), and there are several studies documenting a predisposition of badgers to coexist with Iberian lynx in Mediterranen ecosystems (Fedriani et al., 1999; Garrote & Perez De Ayala, 2019; Palomares et al., 1996), and even increase their abundance in scenarios of lynx rewilding (Jiménez et al., 2019).

We also observed positive associations among the three mesocarnivores (red fox, badger, and pine marten), which were stronger in winter. Both red fox and badger were stronger predictors of pine marten detection rates in winter than land cover variables. Food scarcity and challenging winter conditions may force species to increase the use of similar areas and resources. Previous studies in Scandinavia have reported positive spatial associations among competing mesopredators during times of low prey resources (Cano‐Martínez et al., 2021; Stoessel et al., 2018). Similarly, Monterroso et al. (2020) reported that spatial associations were over five times more frequent than competitive avoidance among mesocarnivores in a Mediterranean ecosystem. They suggested that broad habitat and dietary profile characteristics of mesocarnivores might provide flexibility for potentially competing species to coexist. However, there is evidence that red foxes kill pine martens in northern regions (Lindström et al., 1995), which is probably one of the reasons why pine martens avoid open areas (Storch et al., 1990). On the other hand, the positive association between badgers and red foxes agrees with previous studies, where foxes seemed to seek the company of badgers in the vicinity of setts (i.e., the badger's underground home) (Macdonald et al., 2004). They argued for the possibility that foxes might receive interspecific information from badgers, for example, to find good feeding spots. Also, red foxes and badgers are known to share setts/dens (Macdonald, 1987 as cited in Macdonald et al., 2004), which may indicate a facilitative relationship. Spatial associations between competing species may be facilitated by landscape heterogeneity and by mechanisms of niche separation such as temporal partitioning (Bischof et al., 2014; Lesmeister et al., 2015; Viota, 2012) and small‐scale spatiotemporal patterns (Zalewska et al., 2021). Future research on species activity patterns and temporal overlap could provide a deeper understanding of the mechanisms behind species coexistence.

### 4.3 Contrasting effects of human disturbance

Humans may influence species through direct (e.g., hunting) or indirect (e.g., fear; Ordiz et al., 2013) top‐down processes, but also through bottom‐up mechanisms such as land use and anthropogenic food subsidization. This variety of potential interactions was reflected in the contrasting associations observed in our study area between human disturbance and the detection rates of the different species. Human disturbance was positively associated with the two dominant mesocarnivores (red fox and badger) in summer, which could be related to the use of anthropogenic food resources. Previous studies have documented positive relationships between red fox activity and human settlements (e.g., Jahren et al., 2020; Panek & Bresiński, 2002), although they tend to avoid highly urbanized areas (Červinka et al., 2014). Human population density has been suggested to be a good proxy for anthropogenic food subsidies (Oro et al., 2013), which are used by a variety of generalist carnivores (Manlick & Pauli, 2020), including red foxes and badgers (Gomes et al., 2019; Rosalino et al., 2010). These human resources can potentially increase dietary overlap (Sévêque et al., 2020), which may increase the probability of interspecific competition and intraguild predation in human‐dominated landscapes (Manlick & Pauli, 2020; Newsome et al., 2015). Spatial overlap with humans may be facilitated by an increase in species nocturnality (Gaynor et al., 2018; Lamb et al., 2020). We also expected an indirect negative effect of human disturbance on the smaller mesocarnivore (pine marten) through and increased activity of red fox and badger. Instead, we observed a direct negative association between humans and pine marten in winter, which is similar to previous studies (e.g., Fusillo et al., 2009; Goszczyński et al., 2007), suggesting that pine marten is sensitive to human disturbance.

### 4.4 The effect of seasonality

Based on our predictions, we expected environmental productivity to have a stronger positive effect on carnivore detection rates in winter because of food scarcity (Elmhagen & Rushton, 2007). However, we found a positive association between EVI and some of the carnivores (lynx, red fox, and badger) only in summer. It is likely that land cover productivity did not correlate with food resources during winter due to the presence of snow, which could explain why we did not observe a clear association between EVI and species detection rates in that season. In summer, however, without the presence of snow, the preference for productive areas would be more evident, and it might explain the positive association between lynx and badger. Winter was characterized by stronger positive association among the mesocarnivores, and a lack of association with the large carnivores. This might suggest that, under challenging winter conditions, species may be more influenced by the unpredictability of food resources than by competitive interactions (Stoessel et al., 2018).

We also observed a seasonal difference regarding the effect of agricultural land. This land cover variable was negatively associated with wolf, lynx, and pine marten only in summer, and had no effect in winter. Agricultural fields vary in usage level by humans over the year, with high activity during late spring and summer, and less activity occurring during autumn and winter (Bunnefeld et al., 2006). This might explain the observed seasonal variation and the strong negative effect of agriculture fields in summer for lynx and wolf detection rates. Furthermore, this seasonal variation could also be related to seasonal movements of prey. Moose (*Alces alces*) and roe deer (*Capreolus capreolus*) migrate to areas of lower elevation during winter, in search of higher food availability provided by lower snow depth and artificial feeding sites (Bunnefeld et al., 2006; Mysterud, 1999; Singh et al., 2012). The migration of moose and roe deer to areas of lower elevation during winter could relax the negative effect of fields on lynx and wolf detection rates in winter. Further studies including all four seasons might help determine the effect of specific behaviors constrained to specific seasons, such as mating, reproduction, or human activities such as hunting.

## 5 CONCLUSIONS

Using rich data sets for unintended purposes can result in particular species not being represented equally (Hofmeester et al., 2019). Because we used the same protocol for data collection as Hofmeester et al. (2021), who reported high detectability of wolf, red fox, and badger at lynx‐targeted camera traps, we believe our methods were helpful to evaluate relationships between and among these species.

Our study contributes to a better understanding of the complex ecological processes that influence interactions among carnivores. Land cover variables (bottom‐up) were stronger predictors of mesocarnivore activity than large carnivore activity (top‐down) in Norway. This highlights the importance of considering both bottom‐up and top‐down processes when studying species interactions. It also supports the idea that, in low productive ecosystems with strong seasonality, species appear to be more influenced by the unpredictability of food resources than by the presence of (stronger) competitors. This is also supported by the prevalence of positive associations among species, especially in winter for the mesocarnivores. The relevance of such insights is of special importance in the face of climate change, which will alter seasonal conditions and, consequently, species interactions. The contrasting effects of human disturbance on the different species (benefiting some species while negatively affecting others) show that anthropogenic impacts can be complex and influence multiple trophic levels through different processes. Given that ecological interactions are now happening in a human‐dominated world, there is a clear need to include anthropogenic influences when studying species interactions.

## AUTHOR CONTRIBUTIONS

**Rocío Cano‐Martínez:** Conceptualization (equal); data curation (supporting); formal analysis (lead); investigation (lead); methodology (equal); visualization (lead); writing – original draft (lead); writing – review and editing (lead). **Neri Horntvedt Thorsen:** Conceptualization (equal); data curation (supporting); investigation (supporting); methodology (equal); writing – review and editing (supporting). **Tim R. Hofmeester:** Conceptualization (equal); data curation (supporting); funding acquisition (supporting); investigation (supporting); methodology (equal); writing – review and editing (supporting). **John Odden:** Conceptualization (equal); data curation (lead); funding acquisition (lead); methodology (supporting); project administration (supporting); supervision (supporting); writing – review and editing (supporting). **John Linnell:** Conceptualization (equal); data curation (supporting); writing – review and editing (supporting). **Olivier Devineau:** Formal analysis (supporting); methodology (equal); writing – review and editing (supporting). **Siow Yan Jennifer Angoh:** Investigation (supporting); writing – review and editing (supporting). **Morten Odden:** Conceptualization (equal); funding acquisition (supporting); methodology (equal); project administration (lead); supervision (lead); writing – review and editing (supporting).

## FUNDING INFORMATION

SCANDCAM was funded by the Wildlife Management Fund of the Swedish Environmental Protection Agency as part of the SCANDCAM (NV‐00695‐17 and NV‐2020‐00088) project, the Miljødirektoratet (the Norwegian Environment Agency), the Research Council of Norway (grant 281092), and the Nature Protection Division of the County Governor's Office for Innlandet County, Viken County, Vestfold & Telemark County, Trondelag County, More & Romsdal County, Nordland County, and Troms & Finnmark County. RCM and MO received funding from Inland Norway University of Applied Sciences.

## CONFLICT OF INTEREST STATEMENT

The authors declare no conflicts of interest.

## Data Availability

Data and code are available from the Zenodo repository: https://doi.org/10.5281/zenodo.10553377.

## APPENDIX 1

Posterior median parameter estimates for the final structural equation model (Model 4 in the text) explaining direct associations and interactions of mesopredators (red fox, badger and pine marten) with humans, large predators (wolf and lynx), and bottom‐up variables (Agriculture and EVI), together with their associated 50% (thick blue) and 80% (light blue) credible intervals.

## APPENDIX 2

Posterior median parameter estimates for the final structural equation model (Model 4 in the text) explaining direct associations and interactions of large predators (lynx and wolf) with humans and bottom‐up variables (Agriculture and EVI), together with their associated 50% (thick blue) and 80% (light blue) credible intervals.

## APPENDIX 3

Summary of posterior distributions for the final structural equation model, including the posterior median parameter estimate (Median), the 90% credible interval (90% CRI), the percentage probability of direction (% pd), and the percentage in the Region of Practical Equivalence (% in the ROPE). We have marked in bold *pd* and ROPE values of interest that are discussed.

**Table:**

Response	Parameter	Median	90% CRI		% pd	% in ROPE
Fox	Badger	0.2	0.14	0.25	1	0
Fox	Wolf	0.09	0.04	0.15	**1**	**0.58**
Fox	Lynx	0.06	−0.02	0.13	0.9	0.85
Fox	Humans	0.1	−0.01	0.22	**0.94**	**0.49**
Fox	Agriculture	−0.04	−0.15	0.07	0.71	0.84
Fox	EVI	0.18	0	0.35	0.95	0.22
Fox	Badger:Winter	0.27	0.17	0.38	1	0
Fox	Wolf:Winter	−0.06	−0.14	0.02	0.89	0.8
Fox	Lynx:Winter	0	−0.08	0.08	0.53	1
Fox	Humans:Winter	0.02	−0.05	0.08	0.64	1
Fox	Agriculture:Winter	−0.03	−0.11	0.05	0.73	0.96
Fox	EVI:Winter	0.02	−0.07	0.1	0.62	0.98
Badger	Wolf	0.03	−0.05	0.12	0.74	0.92
Badger	Lynx	0.21	0.11	0.3	1	0
Badger	Humans	0.25	0.11	0.38	1	0.01
Badger	Agriculture	0.26	0.12	0.39	1	0.01
Badger	EVI	0.31	0.06	0.56	0.98	0.06
Badger	Wolf:Winter	0.01	−0.12	0.13	0.54	0.86
Badger	Lynx:Winter	−0.03	−0.13	0.06	0.73	0.9
Badger	Humans:Winter	0.07	0	0.13	0.94	0.81
Badger	Agriculture:Winter	0.13	0.05	0.21	1	0.23
Badger	EVI:Winter	−0.01	−0.16	0.14	0.55	0.75
Marten	Fox	0.06	−0.03	0.14	0.87	0.8
Marten	Badger	0.3	0.2	0.39	1	0
Marten	Humans	−0.07	−0.23	0.09	**0.75**	0.61
Marten	Agriculture	−0.19	−0.38	−0.01	0.96	0.18
Marten	EVI	0.1	−0.2	0.39	**0.71**	**0.39**
Marten	Fox:Winter	0.3	0.17	0.43	1	0
Marten	Badger:Winter	0.13	−0.01	0.28	0.93	0.38
Marten	Humans:Winter	−0.34	−0.74	−0.07	**0.99**	**0.06**
Marten	Agriculture:Winter	−0.05	−0.22	0.12	0.69	0.64
Marten	EVI:Winter	0.09	−0.1	0.3	**0.79**	**0.49**
Lynx	Humans	−0.3	−0.8	0.05	**0.91**	**0.18**
Lynx	Agriculture	−0.53	−0.82	−0.26	1	0
Lynx	EVI	0.49	0.1	0.88	0.98	0.03
Lynx	Humans:Winter	0	−0.43	0.43	0.5	0.33
Lynx	Agriculture:Winter	0.43	0.22	0.65	1	0
Lynx	EVI:Winter	−0.04	−0.26	0.18	0.63	0.55
Wolf	Humans	−1.01	−2.26	−0.05	0.96	0.04
Wolf	Agriculture	−0.95	−1.47	−0.49	1	0
Wolf	EVI	0.39	−0.17	0.99	**0.87**	**0.13**
Wolf	Humans:Winter	0.51	−0.67	1.69	**0.76**	0.09
Wolf	Agriculture:Winter	0.07	−0.44	0.56	0.59	0.27
Wolf	EVI:Winter	0.5	0.05	0.95	**0.97**	**0.05**
